# Nanoscale Bonding between Human Bone and Titanium Surfaces: Osseohybridization

**DOI:** 10.1155/2015/960410

**Published:** 2015-01-15

**Authors:** Jun-Sik Kim, Seok-Man Kang, Kyung-Won Seo, Kyung-Yen Nahm, Kyu-Rhim Chung, Seong-Hun Kim, Jae-Pyeong Ahn

**Affiliations:** ^1^Department of Orthodontics, School of Dentistry, Kyung Hee University, No. 1 Hoegi-dong, Dongdaemun-gu, Seoul 130-701, Republic of Korea; ^2^Department of Orthodontics, School of Medicine, Ajou University, 164 Worldcup-ro, Yeongtong-gu, Suwon 443-380, Republic of Korea; ^3^Advanced Analysis Center, Korea Institute of Science and Technology, 5 Hwarang-ro 14-gil, Seongbuk-gu, Seoul 136-791, Republic of Korea

## Abstract

Until now, the chemical bonding between titanium and bone has been examined only through a few mechanical detachment tests. Therefore, in this study, a sandblasted and acid-etched titanium mini-implant was removed from a human patient after 2 months of placement in order to identify the chemical integration mechanism for nanoscale osseointegration of titanium implants. To prepare a transmission electron microscopy (TEM) specimen, the natural state was preserved as much as possible by cryofixation and scanning electron microscope/focused ion beam (SEM-FIB) milling without any chemical treatment. High-resolution TEM (HRTEM), energy dispersive X-ray spectroscopy (EDS), and scanning TEM (STEM)/electron energy loss spectroscopic analysis (EELS) were used to investigate the chemical composition and structure at the interface between the titanium and bone tissue. HRTEM and EDS data showed evidence of crystalline hydroxyapatite and intermixing of bone with the oxide layer of the implant. The STEM/EELS experiment provided particularly interesting results: carbon existed in polysaccharides, calcium and phosphorus existed as tricalcium phosphate (TCP), and titanium existed as oxidized titanium. In addition, the oxygen energy loss near edge structures (ELNESs) showed a possibility of the presence of CaTiO_3_. These STEM/EELS results can be explained by structures either with or without a chemical reaction layer. The possible existence of the osseohybridization area and the form of the carbon suggest that reconsideration of the standard definition of osseointegration is necessary.

## 1. Introduction

Recently, research on the nanoscale integration of bone with titanium implants has progressed rapidly with high-resolution imaging of biomaterial interfaces with bone. For example, Takatsuka et al. [1] proposed the possibility of chemical integration, since implants with smooth surfaces also demonstrated osseointegration in an animal experimental model. Sul et al. [2] also suggested that chemical integration occurs on the basis of an experimental chemical bonding measurement on the nanoscale osseointegration of a smooth titanium surface to cortical bone. Several* in vitro *experiments [3, 4] showed calcium phosphate precipitation on titanium surfaces in simulated body fluid (SBF). Furthermore, it was recently observed using electron tomography that the interface between a laser-modified surface titanium implant and human bone contains a mixture of oxidized titanium and calcium phosphate on the 100 nm scale [5]. Three-dimensional imaging segments showed direct contact between bone and a titanium implant, but the interaction mechanism was not easy to definitively identify because the removed implants and surrounding bone were fixated, dehydrated, and embedded in plastic resin and then longitudinally cut and polished according to the protocol proposed by Taborelli et al. [6]. This preprocessing of the implant samples could have led to loss of key attributes of the nanoscale osseointegration.

Sample preparation without chemical preprocessing may clarify the integration mechanism of bone and biomaterials at the interface. Scanning transmission electron microscopy (STEM) and EELS methods allow the structural and chemical interactions of the complex interface to be identified, and it would be feasible to analyze the chemical composition of a suspected osseointegrated interface using these techniques. The goal of this study, therefore, was to demonstrate the presence of chemical osseointegration and identify the composition pattern of any chemically integrated layer at the interface using STEM/electron energy loss spectroscopy (EELS) analysis. In particular, we would like to determine whether the oxidized titanium layer of the mini-implant's surface caused a chemical reaction with calcium phosphate of the bone tissue to produce a CaTiO_3_ byproduct, which may then play a critical role in the chemical osseointegration between Ti and bone.

## 2. Experimental

### 2.1. Implants

**Figure 1:**
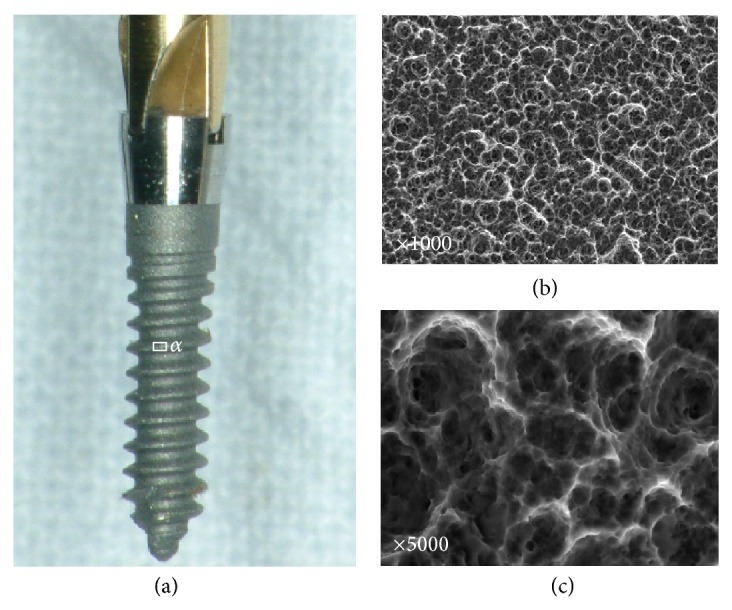
(a) SLA-treated surface of C-implant (ELI Ti-6Al-4Vt). (b) ×1000 magnified SEM view of *α* area. (c) ×5000 magnified view (VEGA II SLH, Czech TESCAN).

The implants used in this experiment were composed of Ti_6_Al_4_V ELI (extra low interstitials) as standardized in ASTM F 136 Rev: 2 (C-implant, Cimplant Co., Seoul, Korea). The specific composition in wt% is Al 6.1%, V 3.9%, C 0.004%, O 0.114%, and Ti bal. The surface was first sandblasted and then acid-etched (SLA treatment). The resulting surface roughness parameters were 1.29 Ry, 9.69 Rt, and 12.08 *μ*m (Figure 1). Third-generation C-implants for temporary skeletal anchorage were selected because of their good osseointegration potential [7–10]. Their combination of good osseointegration potential and easy removal facilitated our study of the chemical osseointegration at the interface in humans.

### 2.2. Scanning Electron Microscopy

The bone-implant contact surface in removed implant samples was studied using SEM. In order to use focused ion beam (FIB) TEM without preprocessing of the samples, the sample thickness should be at most 100 *μ*m, because the FIB cannot illuminate the interface otherwise. In addition, if the sample is too thin, it could be destroyed during interface evaluation. SLA-surface-treated titanium mini-implants were placed and then removed after orthodontic usage. Because a driver is used to remove the mini-implants by unscrewing, the mechanical force could disrupt the integration interface, and consequently it was not easy to find appropriate samples for observation. All removed samples were placed in saline solution and sent to the lab for cryofixation in ethane solution within 1 hour. Then, they were dried at room temperature. Among 100 removed implants, 5 samples were found to have the ideal thickness of bone attachment to satisfy the aforementioned conditions.

### 2.3. Transmission Electron Microscopy

**Figure 2:**
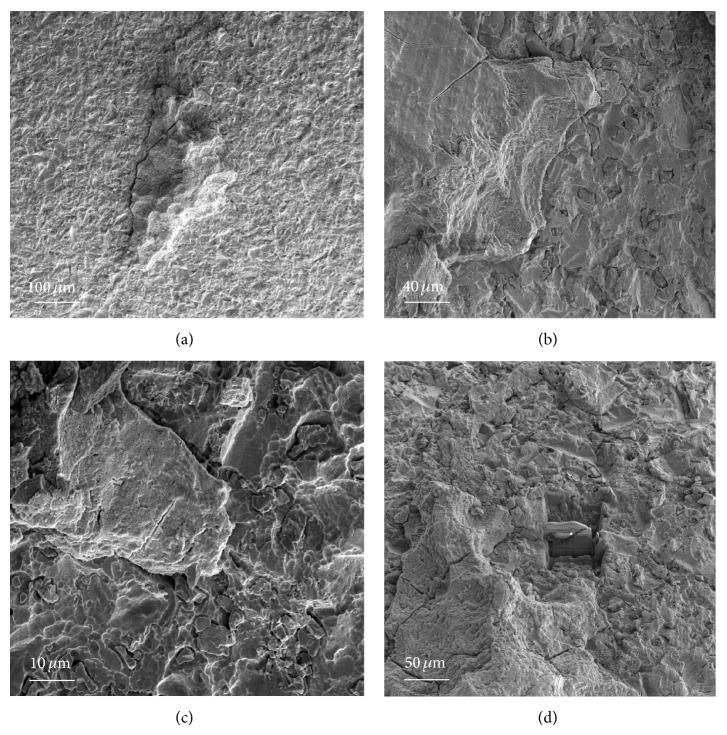
((a)–(c)) SEM images of implant sample. The bone-to-implant interface is clearly maintained upon removal of the implant from patients. (d) A chunk of material with the bone-to-implant contact area was focused ion beam (FIB) milled until totally free from the bulk sample.

The 5 implants were prepared as TEM specimens by SEM FIB (FEI Co.) using an* in situ *protocol [11–15]. The TEM specimen preparation was as follows: rough FIB milling with gallium ions (Ga) at 30 kV at the suspected bone-implant integration surface and then fine milling at 5 kV to minimize the Ga damage. This produces TEM samples <70 nm in thickness, which are attached to a copper grid for study of the atomic composition of the interface between the bone tissue and titanium implant. After this procedure, only 1 implant was found to exhibit intimate integration with bone. This sample was collected after 2 months of use in the mandible of a 23-year-old female patient (Figure 2). The interface structure of this implant was observed by high-resolution TEM (HRTEM). Then, selected area energy dispersive X-ray spectroscopy (EDS) was used to study the atomic composition, and the EDS line profiles were measured simultaneously to determine the changes in atomic composition across the interface. After a full image of the specimen was obtained by a TECANI F20 Cryo STEM equipped with a Gatan GIF866 spectrometer system operating at 200 kV, the region of interest was divided into implant, interface, and bone sections, which were then each observed by EELS. The spectrometer was set to an energy dispersion of 0.05 eV/channel to obtain the best energy resolution at the zero-loss peak (0.9 eV), and the precision of the core-loss peak position (0.05 eV) in the EELS spectra was measured under magic angle conditions for the L2 and the L3 edges of the transition metals (collection angle: 100 mrad for titanium oxides) to avoid anisotropy effects in energy loss near edge structures (ELNESs). The convergence angles were approximately 10 mrad for the Ca, P, Ti, and O spectra.

The EELS spectra obtained in this study were fitted using the ELNES fitting method. This method uses both the edge position and the edge shape to estimate the oxidation state in the compound more accurately and precisely. The validity of this method for the L2 and the L3 edges of transition metals is well established when the absolute peak position must be determined, especially when there are no characteristic peaks. The spectrum of each specimen can be separated as a linear combination of characteristic spectra of reference compounds. Thus, this is a valuable method for using 2D-STEM-EELS spectra to classify the chemical state, atomic symmetry, and oxidation state. In addition, it is useful when corresponding reference data for the analysis of noninteger compounds are available [16]. The interface of interest in this study contains complex noncrystalline compounds. Therefore, because diverse states exist for each component, each spectrum appears overlapped and is different from the reference spectra of crystalline elements. According to previous reports [17], spectra of noncrystalline materials do not have clear peak splitting like crystal spectra. When there are many dispersed elements, as in this study, analysis is often performed with the least-squares linear combination + ELNES fitting method. This combined method was therefore used to analyze the spectrum obtained in this study.

## 3. Results

### 3.1. Imaging and EDS Analysis

**Figure 3:**
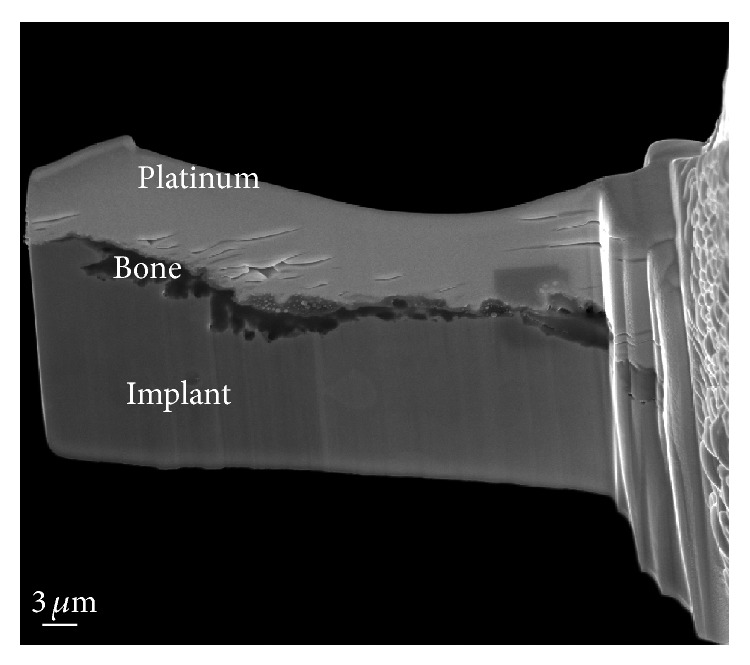
TEM specimen prepared using SEM FIB showing tight binding between bone tissue and the titanium implant.

**Figure 4:**
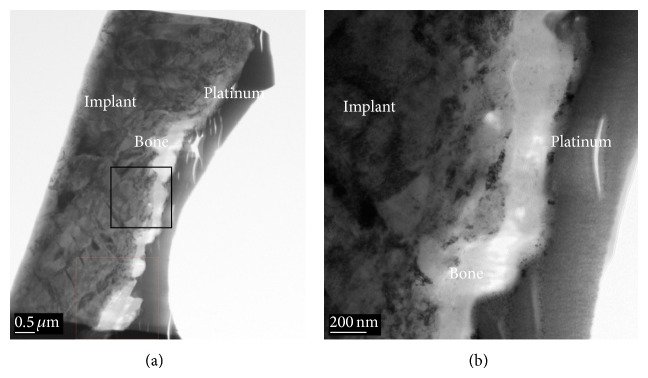
STEM high-angle annular dark-field (HAADF) images of the bone-implant interface at (a) low and (b) high magnification. The nanostructured modified surface oxide layer forms intimate contact between the bone and implant surface. At higher magnification, the bone is seen to be in direct contact with the oxide layer.

**Figure 5:**
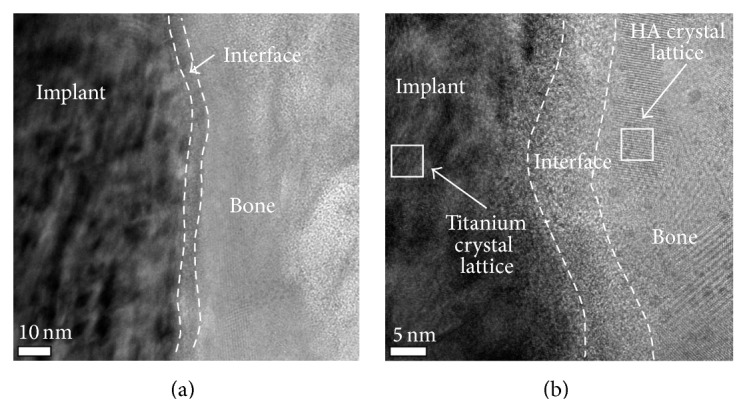
(a) High-resolution TEM images confirming the crystallinity of HA immediately outside of the implant's oxide surface layer. (b) An amorphous interface layer with a thickness of about 20 nm is observed in crystal lattice fringe between titanium and bone.

**Figure 6:**
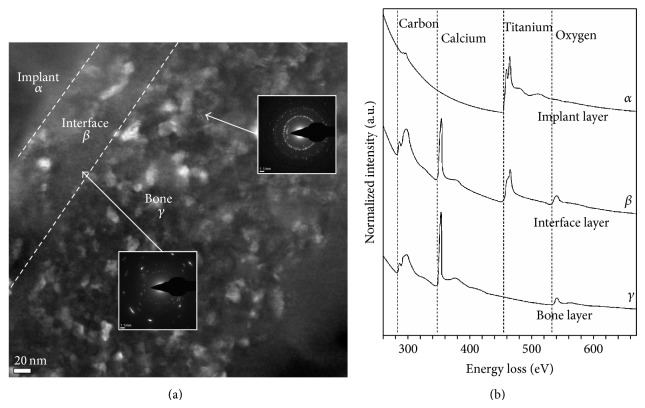
(a) Dark-field TEM images of the crystalized layer and amorphous layer are presented. In the dark-field view, crystalized structures are observed both in the bone and in the amorphous interface layer. And the obtained electron diffraction pattern of each is shown. The crystalized layer pattern matched hydroxyapatite of bone. (b) A spectrum of EELS containing elements of implant, interface, bone layer.

The SEM images obtained prior to implantation in Figure 1 show the microstructural features of the SLA-treated surface. The SEM images in Figures 2(a)–2(c) show that a suitable region of 1 *μ*m in thickness surrounding the interface between the bone and implant surface was obtained. To more closely examine the bone-to-titanium implant interface, FIB was performed and another SEM image was obtained (Figure 2(d)). Thereafter, seven iterations of the ion beam thinning method were performed to prepare the sample for STEM/EELS analysis (Figure 3). The low-power TEM image of the prepared sample in Figure 4 confirms that titanium alloy and bone tissue formed an intimate contact. The lattice fringe in Figure 5 obtained from high-power TEM experiments indicates that the layer between the titanium alloy and bone tissue crystal structures was amorphous. This was also confirmed by dark-field TEM. The existence of hydroxyapatite (HA) within the bone tissue was confirmed by the selected area electron diffraction (SAED) pattern in Figure 6(a). Oxygen, calcium, and phosphorus were also identified at the interface from the EDS line profiles of sample portions including the suspected chemical integration interface. This is consistent with the results of previous studies [5, 18]. While the signal for titanium decreased in intensity from titanium toward the bone tissue, the signals for oxygen, calcium, and phosphorus increased.

### 3.2. TEM/EELS Analysis

**Figure 7:**
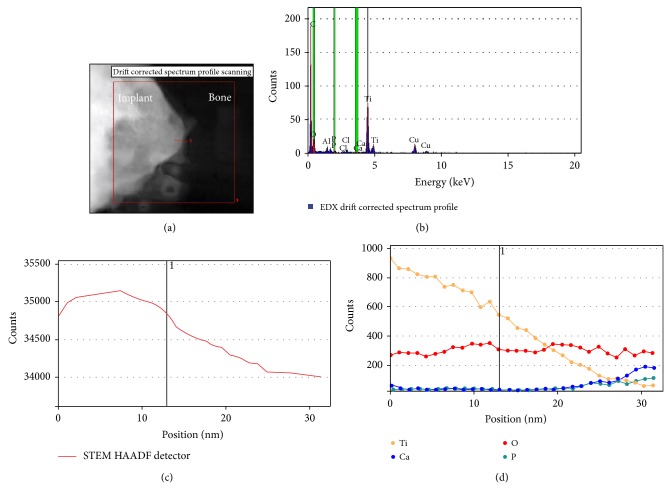
EDS line profile showing that the interface is 10 nm thick and contains oxygen, calcium, phosphorous, and titanium.

The EDS signal for carbon was weak at the interface and strong in the bone tissue. The EELS whole-peak analysis (Figure 6(b)) indicated that only titanium was present in the implant layer, like the EDS results. The EELS spectra also show that carbon, calcium, phosphorus, titanium, and oxygen were all present at the interface. Finally, in the bone tissue, no titanium was observed, but carbon, calcium, phosphorus, and oxygen peaks were apparent. These peak intensity changes in the different areas surrounding the interface were consistent with the EDS line profiles (Figure 7).

**Figure 8:**
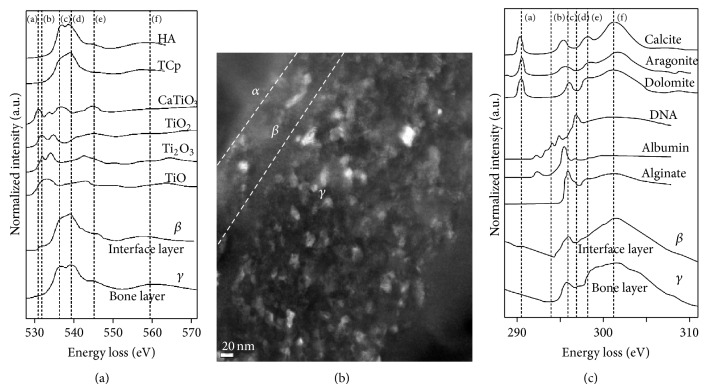
(a) Oxygen K-edge spectra from the image in (b). The oxygen K-edge spectrum of the interface layer shows the same apex as that of TCP. The oxygen K-edge spectrum of the bone layer shows the same apex as that of HA. (b) Image of the interface region, where *α* is the implant layer, *β* is the interface layer, and *γ* is the bone layer. (c) C K-edge spectra of the image in (b). The shape of our observed C K-edge is similar to that of alginate.

Two well-defined peaks were observed for titanium in the implant layer, but this split was not well-defined in the interface layer, and the peak was smaller because the titanium existed in a more amorphous state and not as crystalline titanium dioxide (Figure 8). This loss of the high energy titanium oxide peak at the interface is clear evidence of chemical modification. Proceeding from the interface to the bone tissue, peaks for calcium and phosphorus increased in intensity without drastic shape changes, proving the existence of these elements in both bone and interface.

**Figure 9:**
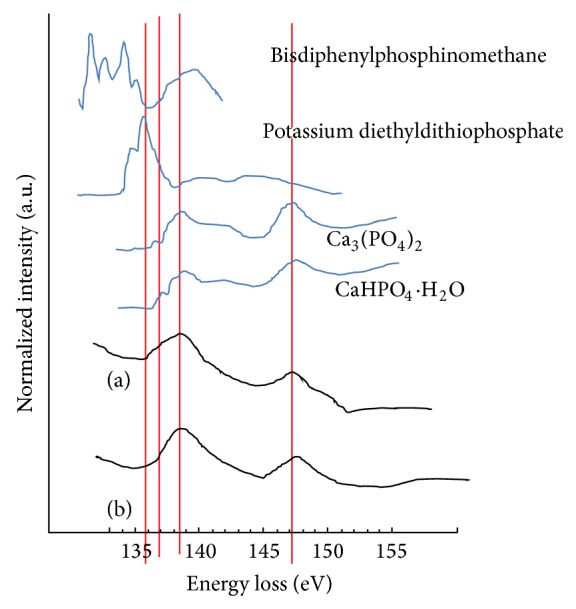
Phosphorus L-edge spectra of (a) the interface layer and (b) the bone tissue layer. The P L-edges have peak positions and shapes similar to those of calcium phosphate in both layers. However, we cannot differentiate between tricalcium phosphate and hydroxyapatite from the P L-edge.

The phosphorus spectrum has a peak pattern similar to that of calcium phosphate in the mixed layer and bone tissue layer (Figure 9). In this study, it was not possible to distinguish tricalcium phosphate (TCP) and HA by P-edge analysis because of the similarity of their patterns, so they were differentiated by O K-edge analysis instead (Figure 8(a)). The oxygen spectra from the interface and bone tissue layer showed double peaks in the interface close to the titanium and several peaks with a shoulder in the interface close to bone. These peaks, however, were different from those of pure oxygenated titanium types, especially in terms of the location, shape, and separation distance of the peaks. The TCP-like signal in the oxygen spectrum with anterior and posterior shoulders grew bigger at the midpoint of the interface, and the sharp upper spikes clearly distinguished this material from HA. The anterior and posterior shoulders disappeared in the bone tissue, and a flattened summit resembling an HA signal appeared (Figure 8(a)). Comparison of the ELNES of the oxygen spectrum at the interface obtained in this study and the reference spectrum suggests the existence of TCP, titanium oxide, or calcium titanate in the interface.

The carbon spectrum in Figure 8(c) has one sharp anterior peak and one broad posterior peak. The interface and bone tissue layer share the same carbon spectrum shape. The similarity of the peak pattern in an alginate spectrum and in our data suggests that the carbon in the mixed layer and in the bone tissue layer was in the form of polysaccharides.

**Figure 10:**
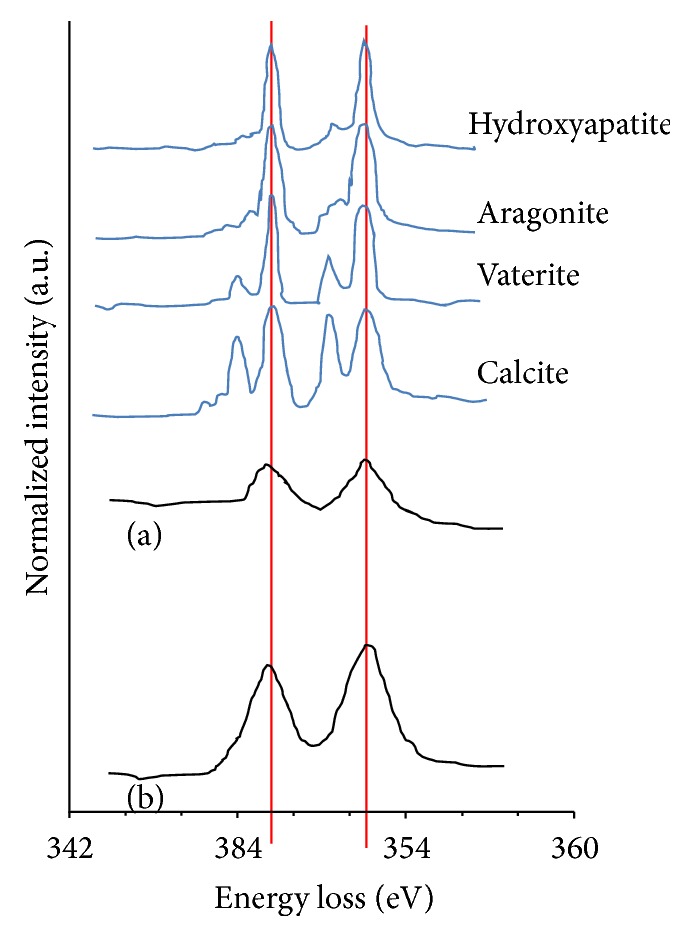
Calcium L-edge spectra of (a) the interface layer and (b) the bone tissue layer. The Ca L-edges have peak positions and shapes similar to those of hydroxyapatite rather than other reference compounds in both layers.

In the calcium L-edge spectrum, because the positions of the reference peaks and the L3/L2 peaks were identical and no splitting of the peaks was observed, the existence of HA or TCP can be assumed. Carbonates including calcite were ruled out by the dissimilarity of the peak patterns (Figure 10).

## 4. Discussion 

In this study, we simultaneously applied two major different approaches to perform a precise nanoscale study of bone-to-implant contact. First, we used human orthodontic mini-implant samples. Unlike prosthetic implants, orthodontic mini-implants are used as temporary skeletal anchorages in orthodontics and are removed at the end of usage. This approach allowed us to illuminate the nanoscale interactions at bone-to-implant contacts in humans instead of in experimental animal models.

Second, we prepared samples without any chemical pretreatment, unlike previous studies. Such chemical pretreatments could create artifacts in the structures of the interface at the bone-to-implant contact. In order to bypass the pretreatment of samples, the samples were prepared by cryofixation and sliced using* in situ *SEM FIB protocols for TEM observations. After this preparation, STEM-EELS analysis could successfully determine the atomic chemical composition and structure on the nanometer scale.

The specimen thickness generally should be less than 50 nm for EELS analysis [19]. However, during STEM observations, the concentrated electrons in a narrow specimen could cause specimen distortion or fracture, so too thin specimens can be a problem [20]. Furthermore, biological specimens like the bone specimen used in this study are weaker than inorganic materials like minerals and metals, so thicker slices have usually been used. Therefore, specimens with thicknesses of about 70 nm, slightly thicker than the usual EELS specimens, were observed. For thicker specimens, peak splitting is not noticeable and the effects of the crystalline direction are lost. The electrons used to scan the specimen in STEM-EELS analysis have very small masses, so the peaks are greatly influenced by the surrounding environment. Thus, maintaining a steady environment and avoiding instrumental vibrations are critical during observation. Furthermore, the consistency of the electronic beam energy is not stable, so it is necessary to find the absolute position of the ionization threshold for calibration. Usually, automatic computer calibration of the zero-loss peak is performed before observation of a specimen [21]. However, zero-loss peak control was not performed in this study, so the ionization threshold or peak locations were not determined on an absolute scale. Moreover, for the observation of peak splitting and more specific determination of the ELNES, 0.01 eV energy resolutions are needed. However, only 0.2 eV energy resolution was achieved in this study, which makes the observation of peak splitting difficult.

There are six ways to analyze EELS spectra: the white-line ratio method, chemical shift method, O K-edge onset method, edge onset difference method, two-parameter method, and ELNES fitting method [16]. The least-squares linear combination and ELNES fitting method was used to analyze the EELS spectrum in this study, as discussed above. This allowed each element's form and abundance to be studied from the titanium surface toward the bone.

First, because the implant layer exhibits only the two characteristic peaks of titanium [22] and no carbon, calcium, phosphorus, or oxygen (Figure 6), carbon, calcium, and oxygen peaks appearing in the other layers are confirmed not to be caused by contamination during the specimen preparation by SEM-FIB. Next, the Ti L2- and L3-edge EELS spectra can be used to identify the structure in metal titanium and titanium oxides, because the split between the L3 and L2 peaks increases and the centers of the L3 and L2 peaks are chemically shifted to higher energy losses as the degree of oxidation increases. The TiO_2_ peaks are split into four peaks, whereas metal titanium had just two peaks because the degeneracy of the 3*d* orbital of Ti is increased by the electrical field of the oxygen atoms. Amorphous TiO_2_ shows a split of 1.6 eV, which is less than the 2.5 eV split observed in crystalline TiO_2_ because of the weaker electrical field of the oxygen atoms in the amorphous state [22].

Based on these considerations, our study suggests that titanium oxide may exist in the interface because the peaks of the Ti L2- and L3-edge were split and the centers of the peaks moved toward higher energy loss as the investigated area moved from the implant layer to the interface. In particular, the peak splitting of 1.6 eV is less than that of crystalline titanium oxide, which suggests that amorphous TiO_2_ exists at the interface (Figure 6(b)). This conclusion is consistent with the dark-field TEM image in Figure 6(a).

To further identify this structure, we note that Kourkoutis et al. [22] found that the structure of the Ti L2- and L3-edge was sensitive to the valence of titanium in titanium oxides (Ti^4+^) or perovskite (Ti^3+^), although changes in the A-site cation in Ti^4+^ compounds had very little effect on the edge. In our study, we could not determine whether titanium had the perovskite structure or the titanium oxide structure because the Ti L2- and L3-edge of titanium showed changes between the implant layer and the interface but no changes in the interface itself.

Similarly, we note that the edge of Mn^2+^/TiO_2_ titanium is known to be sharper than that of clean TiO_2_, because the L-edge peak is widened when the octahedral symmetry is bent toward tetragonal symmetry, as in rutile TiO_2_, but narrows for a trigonally distorted octahedral structure (FeTiO_3_) [23]. Therefore, a relatively sharp L-edge peak could be a proof that MnTiO_*x*_ was formed, although broad peaks can still appear when thick Mn is present on the sample surface. As such, the reason why no sharp Ti edge was observed in the presence of the perovskite structure in our study might be the amorphous perovskite structure, the thickness of the specimen, or the low resolution.

As the valence of Ti in titanium oxide increases, the oxygen edges take complex shapes, just like the Ti L2- and L3-edge [24]. For convenience, the oxygen edge peaks are named from low energy loss to high energy loss as A (A1 and A2), B (B, B^*^, and B0), C, and D. For TiO (Ti^2+^), only very simple A and B peaks appear. Ti_2_O_3_ (Ti^3+^) shows splitting of A peak into the lower intensity A1 and higher intensity A2. Rutile TiO_2_ (Ti^4+^) shows a split of 2.7 eV between A1 and A2, which are sharp and have the same intensity, and a B peak with clear anterior and posterior shoulders. The split between the A1 and A2 peaks is due to the energy difference between the *e*_g_ and *t*_2g_ molecular orbitals, which is sensitive to the oxidation degree of titanium. The split is 1.5 eV in TiO, 2.2 eV in Ti_2_O_3_, and 2.7 eV in TiO_2_.

In this study, the composition could not be inferred by comparing the ELNESs because many kinds of matter were mixed up at the interface, and the ELNESs of the oxygen spectra of each component overlapped. However, the distance between the A1 peak and B peak in the oxygen K-edge spectrum is known to decrease from 12 eV for titanium oxide as the oxidation degree of Ti decreases [15]. Therefore, the distance between the anterior and posterior shoulders of 13 eV in our data suggests that other elements including oxygen indeed existed at the interface, in addition to the titanium oxide.

In calcium phosphates, all oxygen K-edge spectra have two major signals centered at 538 eV and 557 eV [25], along with a weak prepeak at 531 eV and a shoulder at 544 eV. The signal centered at 538 eV consists of two major overlapping peaks centered at 536 eV and 539 eV for HA, whereas there is no 536 eV peak for TCP. Therefore, the signal at 536 eV can be regarded as the chemical fingerprint of oxygen in the HA structure, which provides an additional electron state above the Fermi energy that is not present in TCP (536 eV). In particular, it is presumed that the OH (or oxyl group) in HA is the origin of this additional state. The other signals in the oxygen K ionization edge spectra are associated with the different crystal structures of the two compounds. For example, the crystal symmetry of TCP is rhombohedral, with a Ca cation and PO_4_ tetrahedral units. Thus, all oxygen atoms are restricted to the PO_4_ tetrahedra in TCP. In contrast, HA contains a hydroxyl (or oxyl) group as well as a PO_4_ unit and Ca cations and thus has an additional sublattice oxygen site. Finally, we note that there is a 13 eV separation between the very weak prepeak and the posterior shoulder, a 9 eV separation between the peak onset at 535 eV and the posterior shoulder, and a 5 eV separation between the highest peak at 539 eV and the posterior shoulder [25].

In this context, the single sharp peak in the posterior part of the apex of the upper portion of the oxygen spectrum from the interface suggests that the calcium phosphate in the interface exists as TCP rather than as HA. In addition, the separation of 13.1 eV between the distinct anterior shoulder of the oxygen K-edge from the interface and the posterior shoulder and the 6.5 eV separation between the highest peak and the posterior shoulder imply that other elements including oxygen exist within the interface, in addition to calcium phosphate.

The P L-edge spectrum has a similar peak position to that in the reference phosphorus spectrum of calcium phosphate [23, 26]. However, we cannot distinguish between TCP and HA using the P L-edge spectrum because of its low resolution. Our study's Ca L-edge peak was similar to HA or aragonite reference peaks [27], but the low resolution again prohibited accurate classification. Finally, among the main cellular carbon K-edge peaks at 285.2 eV for aromatic groups of proteins, 286.8 eV for phenolic or ketone groups of polysaccharides, and 288.2 eV for carbonate groups [21], the C K-edge spectrum in this study is most similar to that of an alginate polysaccharide, as seen in Figure 8(c).

In the bone area, the oxygen K-edge spectrum has wide peak representative of HA and no anterior shoulder like that seen in the spectrum from the interface. Clearly, common calcium phosphate compounds exist in the bone tissue layer [25]. Moreover, the distance between the posterior border of the peak and the posterior shoulder is smaller than 6.5 eV, indicating that the elements that caused the anterior shoulder and posterior shoulder observed at the interface are not present in the bone tissue layer. Heimann and Wirth insisted that the prepeak signal they observed in the oxygen K-edge spectrum was caused by chemical reduction by the electron beam [28]. However, no such prepeak was found in the bone layer, which confirms that no oxygen gas was produced by reduction by the electron beam in this study.

Other alterations of the spectrum by the electron beam or ion beam are also possible, such as reduction of the Ti^4+^ in TiO_2_ when oxygen is removed by Ar surface sputtering [23]. Depending on the sputtering time, the oxygen edge and the titanium edge can both change. For example, sputtering decreases the *e*_g_-*t*_2g_ energy difference of the oxygen edge from 2.7 eV to 1.8 eV and causes the 555 eV peak to disappear. The titanium edge spectrum changes from 4 peaks to 2 broad peaks, and the center of the peak shifts to lower energy. Reduction by the electron beam lowers the titanium oxidation state, which affects both the oxygen and titanium edges. The splitting of the titanium edge peak is reduced, and the peak is shifted to lower energy. No clear splitting was observed in our study, which could be due to such reduction of the titanium oxidation state during the Ar ion milling for TEM sample preparation or reduction by the electron beam.

Bone tissue has collagens composed of carbon, so carbon is obviously found in the EELS spectrum of the bone tissue sample. During the TEM specimen preparation by SEM-FIB milling, carbon from the bone tissue could be deposited in subsequent layers along the cutting direction. However, any such contamination was removed by ion milling in this study. In particular, no carbon was found in the implant layer, as would be expected if such contamination was present. In addition, if cutting contamination occurred, the white gold from the surface would have appeared in all layers, in contrast to our results. Thus, the presence of a carbon peak from the interface implies the actual presence of carbon atoms. When calcium phosphate binds to the titanium surface, elements like carbonate and magnesium prevent the crystallization of calcium phosphate [29]. Therefore, the carbon in the interface may be predicted to originate from the carbonate. However, the carbon and calcium EELS spectra from the interface suggest that carbon originated from polysaccharides rather than carbonate.

In an effort to clarify the role of carbon in this area, an animal experiment was implemented. The same titanium implant was used in beagle dogs and RNA sequencing was performed on the collected samples. The implant samples from the early osseointegration stage revealed an extracellular matrix- (ECM-) related gene and therefore confirmed the presence of carbon in the mixed area, as observed in our EELS results.

Given these considerations, the most important aspects of the oxygen K-edge spectrum are the outer shape and the anterior/posterior shoulder of the peak, which arise from the overlapped spectra of oxygen in different compounds. The distance between the prominent anterior and posterior peaks is within 12 eV in titanium oxide and 13 eV in calcium titanate [30]. The 13 eV separation between these peaks in the oxygen spectrum from the interface indicates the possibility that calcium titanate is present. Otherwise, there are several possible origins for the anterior shoulder of the oxygen spectrum. Hydrogen-containing groups such as OH or H_2_O were ruled out because their peak positions [30] are 10 eV anterior to the main peak threshold. Transient oxygen gas produced by the electron beam [31] was also ruled out because there was no anterior shoulder in the spectrum obtained from the bone tissue layer under the same observation conditions. Calcium phosphate with a self-induced prepeak was rejected because of the weak intensity of this peak [30]. The posterior shoulder peak could be due to calcium phosphate itself [25]. For example, a peak appeared 5 eV away from the main peak in Gregori's experiment [25], and our data showed a peak 6.5 eV away from the main peak. Therefore, the oxygen K-edge spectrum of our interface may indicate the presence of calcium titanate. The oxygen spectra of titanium oxide and titanium phosphate overlap the spectra of calcium titanate and calcium phosphate, so the specific origin of this signal cannot be determined from our oxygen spectrum.

This study brings into question the conventional definition of osseointegration as direct micromechanical bone-to-implant contact. In particular, our result shows that, at least in the early stage of osseointegration, samples examined without preprocessing contain amorphous osseohybridization zones between the bone and implant that contain carbon in the form of polysaccharides in a low density.

The sample used in this study was cryofixed and minimally pretreated by SEM-FIB, but drying was an essential part of this preparation, so the hydrated* in vivo* environment was not reproduced. Therefore, further study in a cryogenic FIB/TEM environment should be conducted. The ELNESs of Ti, Ca, and other elements determined in this study were limited, since the resolution of our instruments was not very high. Higher resolution instruments are needed for more accurate analysis. Finally, the collected implants were maintained intraorally for only 2 months in this study, and further long-term study is required to confirm the existence of osseohybridization zone in long-lasting implants.

## 5. Conclusion

Whether the chemical reaction layer exists or not, intimate bonding between titanium and bone was confirmed, and we found that the calcium phosphate close to the titanium in the bone tissue layer resembles TCP rather than HA. Therefore, if there is no chemical reaction layer, it can be concluded that the amorphous TCP is bound to the titanium oxide layer of the titanium implant by electrostatic force. If the chemical reaction layer is present, it is presumed to consist of CaTiO_3_. Interestingly, there is carbon near the implant surface in the form of polysaccharides and not carbonate. The RNA sequencing data suggest that the carbon is derived from the ECM. Besides the confirmation of nanoscale osseointegration, an osseohybridization zone of mixed composition appearing in the early stage of osseointegration was discovered, and further study is needed on this finding.
